# Enhanced output performance on LbL multilayer PVDF-TrFE piezoelectric films for charging supercapacitor

**DOI:** 10.1038/s41598-019-43098-6

**Published:** 2019-04-29

**Authors:** Moon Hyun Chung, Seunghwan Yoo, Hyun-Jun Kim, Jungjoon Yoo, Seol-Yee Han, Kyung-Hwa Yoo, Hakgeun Jeong

**Affiliations:** 1 Energy ICT·ESS Laboratory, Energy Efficiency Technologies and Materials Science Division, Korea Institute of Energy Research, 152 Gajeong-ro, Yuseong-gu, Daejeon 34129 Republic of Korea; 2 Department of Physics, Yonsei University, 50 Yonsei-ro, Seodaemun-gu, Seoul 03722 Republic of Korea; 3 Separation and Conversion Materials Laboratory, Energy Efficiency Technologies and Materials Science Division, Korea Institute of Energy Research, 152 Gajeong-ro, Yuseong-gu, Daejeon 34129 Republic of Korea

**Keywords:** Devices for energy harvesting, Devices for energy harvesting, Supercapacitors, Supercapacitors

## Abstract

The piezoelectric nanogenerator (PENG) has the potential to become a promising power supply for monitoring and sensors in Internet of Things (IoT) systems through wireless networks. In order to further increase the utilization of energy harvesters in an IoT system, we introduce a novel approach that greatly enhances the piezoelectric output performances by employing the layer-by-layer (LbL) method. Poly(vinylidenefluoride-co-trifluoroethylene) (PVDF-TrFE) polymer film, which has piezoelectric properties and mechanical flexibility, was used for the active layer in PENG. The maximum open-circuit voltage and closed-circuit current of the LbL multilayer PENG reached 34 V and 100 nA, respectively. In particular, the closed-circuit current of the LbL multilayer PENG was dramatically improved to be five times higher than that of the single-layer PENG. Furthermore, a supercapacitor was employed to investigate the energy storage capability of PENGs using different methods. The proposed LbL multilayer PENG is expected to be a candidate for a promising power supply for self-powered systems in the IoT system.

## Introduction

A variety of sensors is used in the environment for measuring temperature, relative humidity, gas, motion, light, and so on. The need for sensors has increased more and more with the development of Internet of Things (IoT) technologies. Ubiquitous sensor networks, which are wireless sensor networks, are receiving much attention these days for promising IoT technology because they can obtain information from the local area and communicate with each other. As the area where sensors are used becomes wider, however, problems occur in the power supply with regard to battery replacement and wire connections. To overcome these shortcomings, one is that there are some methods that use sustainable energies such as solar, wind, thermal, and vibration. In particular, mechanical energy such as electromagnetic^1,2^, piezoelectric^3–5^, and triboelectric^6–8^ has been gathered mainly from human bodies and specific environments in vibration form. Among sustainable energies, the piezoelectric nanogenerator (PENG) has been noted as an efficient energy harvester of mechanical energy in the surrounding environment^9,10^. The other is a supercapacitor that is alternative to a primary battery. Briefly, supercapacitor is widely studied for one of the energy storage devices^11–14^ because it has advantages of fast charge-discharge rate, high power density, long term stability, superior rate capability, and low cost^15,16^. To operate sensors with the help of supercapacitor, employing energy harvesting which can supply the power consistently is useful because supercapacitor needs energy sources that would be stored in^17^.

Many piezoelectric materials, which are inorganics (*i*.*e*. PZT^18^, BaTiO_3_^19^, ZnO^20^ etc.), generally have a high piezoelectric constants, high permittivity and ferroelectric properties. But it has a disadvantage of stiffness and brittleness. Organic materials (*i*.*e*. PVDF^21^, PVDF-TrFE^7^, PDMS^22^ etc.) could be a solution because it has flexibility and exhibits good piezoelectric performance.

Working a sensor device generally needs low voltage and high current. But a sensor network connected many sensor devices requires high voltage for operating system. However, piezoelectric energy harvesting, which has high impedance, generates a high voltage but low current. Therefore, additional components^23–26^ or structural transformation^27,28^ is needed in order for a piezoelectric energy harvester system to produce a high current. Multilayer structures, one type of structural transformation, can increase the current and reduce the impedance^29^. In addition, the PENG could have durability that endures fractures even under vibration or bending conditions. Therefore, the organic materials have been actively applied to applications such as tactile sensors^30,31^, pressure sensors^32^, and power harvesting applications^33^.

In this paper, we fabricated PVDF-TrFE multilayer film in order to increase the closed-circuit current and open-circuit voltage. By employing Cu tape simply between PVDF-TrFE multilayers, an LbL multilayer PENG was also fabricated and compared with a single-layer PENG. X-ray diffraction (XRD) measurement was performed to investigate the crystallinity of the PVDF-TrFE. Finally, we examined the capacitance of piezoelectric films with supercapacitor charging under bending conditions.

## Results and Discussion

**Figure 1:**
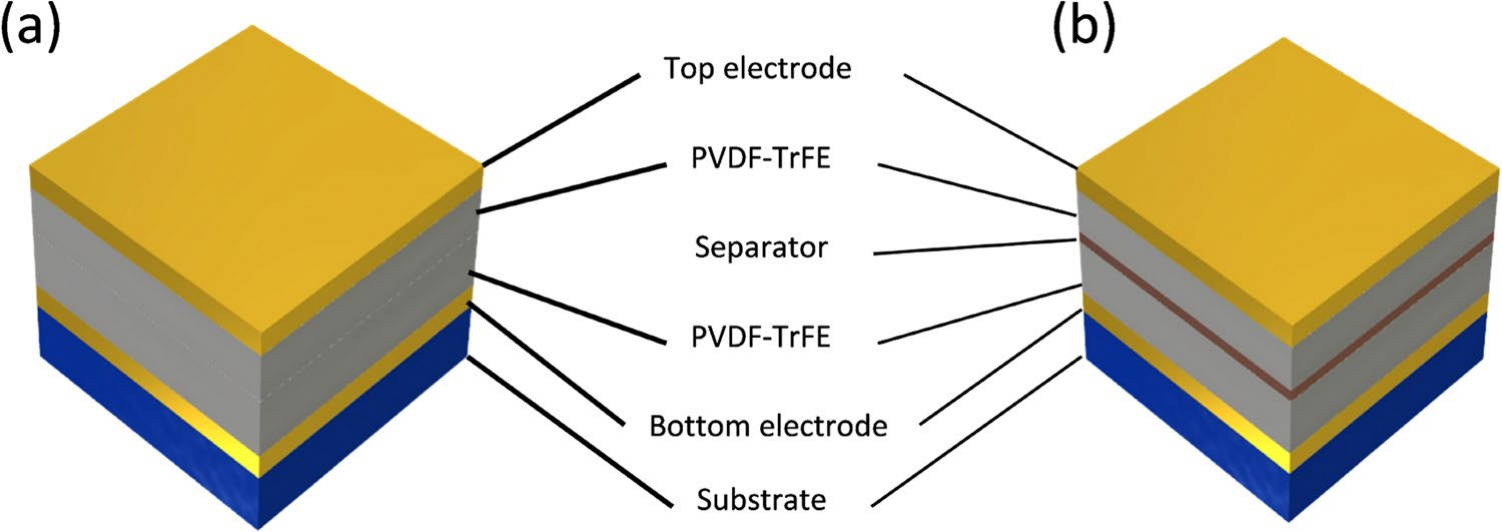
Illustration of piezoelectric multilayer structures: (**a**) stack-up multilayer PENG and (**b**) layer-by-layer (LbL) multilayer PENG.

To increase the electrical performance of the multilayer energy harvester, a simple method was adopted to build PVDF-TrFE multilayer structures such as stack-up and layer by layer. Figure 1 illustrates a piezoelectric PVDF-TrFE multilayer structure. A stack-up multilayer is composed of two PVDF-TrFE films as active layers and Au electrodes. A Layer-by-Layer (LbL) multilayer was prepared by the following method. First, a 160-nm-thick Au bottom electrode was deposited on a flexible substrate. PVDF-TrFE film was spin-coated on the bottom electrode annealed on a hot plate. To fabricate the LbL multilayer, a separator of Cu tape was attached on the first active layer of the PVDF-TrFE film. The Au electrode was deposited as a separator electrode but is not suitable for this role. When the PVDF-TrFE solution was coated for the second active layer on the Au separator electrode by spin-coating, the Au electrode was not clearly coated. The PVDF-TrFE solution had poor dispersion with the Au electrode. Therefore, Cu tape was chosen as a separator electrode owing to its simplicity and good compatibility with PVDF-TrFE. Then, the PVDF-TrFE solution was coated again on the Cu tape for the second active layer. The LbL multilayer was connected in series with the top and bottom electrodes.

**Figure 2:**
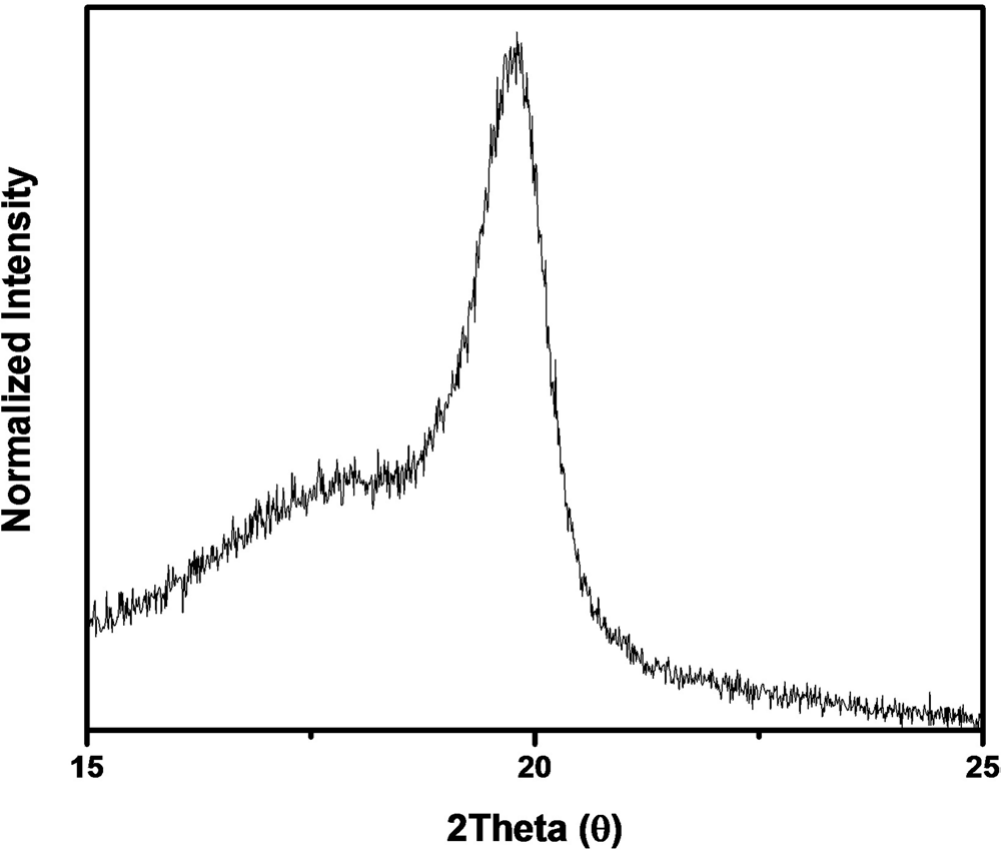
XRD result of β-phase of PVDF-TrFE film.

**Figure 3:**
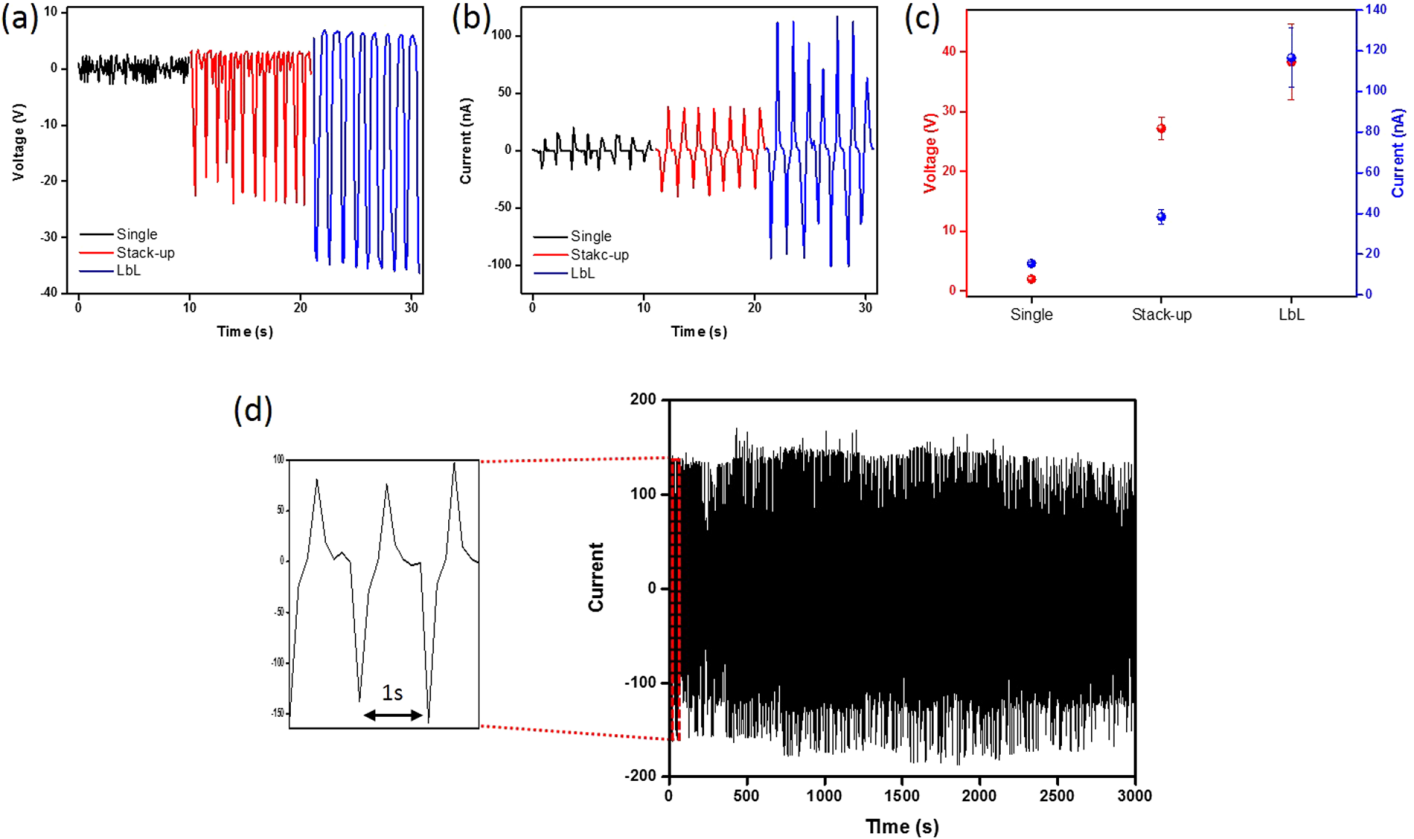
Open circuit voltage and closed circuit current of PVDF-TrFE energy harvester. Measured (**a**) open circuit voltage and (**b**) closed circuit currents of the single layer, stack-up multilayer, LbL multilayer, respectively. (**c**) Compare to open circuit voltages and closed circuit currents with single layer, stack-up multilayer, LbL multilayer and (**d**) durability test of LbL multilayer PENG during 3000 cycles under repeated bending cycle at frequency of 1 Hz.

To confirm the PVDF-TrFE crystalline phase, XRD analysis was carried out. As can be seen in Fig. 2, the XRD peaks of the PVDF-TrFE exhibit an intense β-phase peak diffraction at an angle of 2θ at around 19.8º, which indicates the presence of the pyroelectric phase^34^. Figure 3 shows the output performance of PENGs with single layer, stack-up, and LbL multilayer, respectively. The output voltage and closed-circuit current were measured under repeated bending at a frequency of 1 Hz. The single-layer PENG generated an output voltage of 2.3 V and closed-circuit current of 15.4 nA as shown in Fig. 3(a,b). This result is similar to that of other research^35^. However, the output performance of the single layer was not sufficient to charge the supercapacitor for IoT sensor network systems. Thus, we tried to find other methods such as the stack-up structure and layer-by-layer structure. The stack-up multilayer PENG generated output voltage of 25 V and closed-circuit current of 38 nA. The electrical output performance of the stack-up multilayer PENG increased but it was not dramatically improved because of the internal resistance increment with the thickness of the active layers^36^. The output voltage and closed-circuit current of the stack-up multilayer PENG increased by 10 times and were 2 times higher than those of the single layers, respectively. Although the electrical output performance was improved in the stack-up multilayer PENG, it was still not sufficient for charging a supercapacitor owing to a low closed-circuit current. Therefore, we used Cu tape as a separator to decrease the internal resistance between the PVDF-TrFE layers. The LbL multilayer was prepared in the same way as a stack-up multilayer except that a Cu tape separator was added.

**Figure 4:**
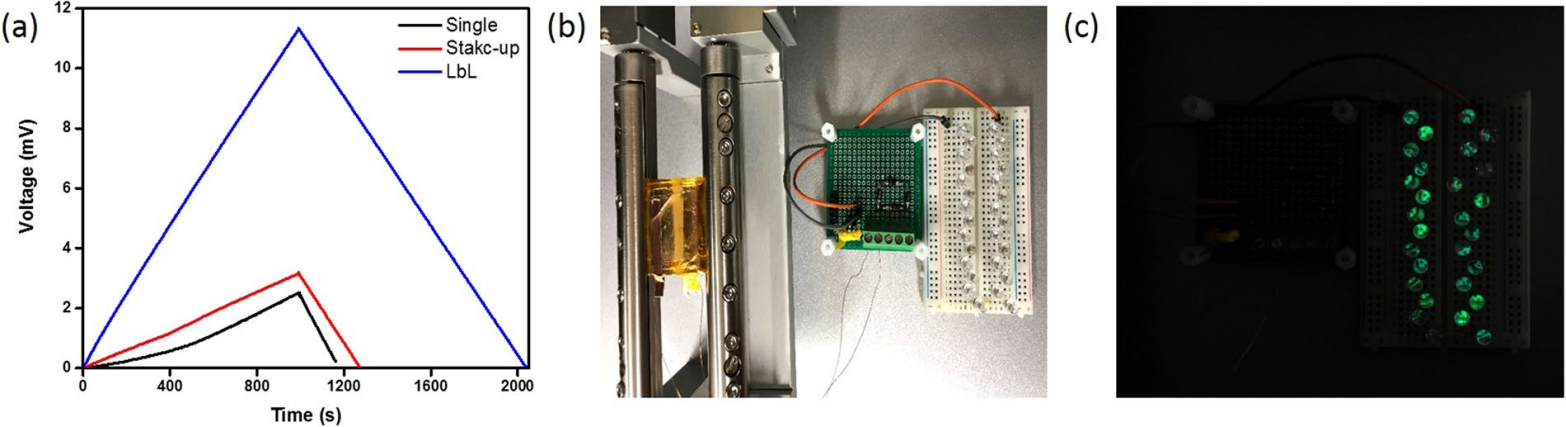
(**a**) Charging-discharging performance of PENGs in chip-type supercapacitor for energy harvesting with constant discharge current density, snapshots of instantaneous lighting of 28 green LEDs in series with LbL multilayer PENG (**b**) before and (**c**) during moment of bending cycles.

The expected benefits of the Cu-tape separator electrode for LbL multilayer were an increase in the pathway and helping the dipoles to align with the electrical field^37,38^. As the number of layers was increased, the output voltage and closed-circuit current increased compared to those of a single layer. For the LbL multilayer PENG, the highest values of electrical properties indicated an output voltage and closed-circuit current of approximately 34 V and 100 nA, respectively, under a bending state. This was over 13 times and 6 times higher than those of the single-layer PENG, respectively. As shown in Fig. 3(c), this tended to enhance the electrical performance of the multilayer owing to increased capacitance^39^. Table 1 lists the capacitances of PENGs of single, stack-up, and LbL types. An increase in capacitance could cause an increase in electrical output performance. And we measured LbL multilayer of the variety of external load resistance. The value of voltage and current can reach the 33 V and 310nA, respectively. The power density shows the 7.8 mW/m^2^ at 100 MΩ (Fig. S1(a,b)). To estimate the electrical and mechanical stability of the LbL multilayer further, we performed cycling over 3000 times (Fig. 3(d)). As for the charging-discharging performance, Fig. 4(a) shows the measured voltages of the chip-type supercapacitor for energy harvesting as charged by each of the PENGs. The charging speed was enhanced by about four times in the LbL multilayer PENG compared to the single-layer PENG because the LbL multilayer PENG showed a higher value of current density than the others. The discharge also follows the same trend as charging. The LbL multilayer PENG with outstanding electrical output performance could operate as a self-powered electric device using a full-bridge rectifier and electrical circuit system (Fig. 4(b,c)). During the bending cycle with the LbL multilayer PENG, 28 green LEDs were turned on immediately. The LbL multilayer PENG could be a candidate for a real-time self-powered generator that is suitable for sensor device applications.

**Table 1:** Capacitance measurement of PENGs with single layer, stack-up multilayer, and LbL multilayer.

	Single	Stack-up	LbL
Capacitance (nF)	12.614	12.627	13.482

## Conclusions

To increase the output voltage and closed-circuit current, we successfully demonstrated an LbL multilayer PENG based on PVDF-TrFE film. Through the presence of Cu tape as a separator electrode between two active layers, the piezoelectric electrical properties of the output voltage and closed-circuit current were effectively improved. The output voltage and closed-circuit current of the stack-up multilayer PENG were improved by 10 times and were 2 times higher than those of the single layer, respectively. Although the electrical output performance of the stack-up multilayer PENG was also enhanced, it produced an insufficient level of current. With the utilization of Cu tape as a separator electrode, the electrical output performance was effectively enhanced. The resulting LbL multilayer generated an output voltage and closed-circuit current of about 34 V and 100 nA, respectively. And the power density showed the 7.8 mW/m^2^. These were improved by 14 times and were 6 times higher than those of the single layer. As for charging a supercapacitor with PENGs, the charging speed of the LbL multilayer PENG was improved by about four times that of the single-layer PENG. The results show that the LbL multilayer PENG can be sufficient for charging a supercapacitor efficiently. We hope that the LbL multilayer PENG can be used as a self-powered generator for wireless sensor networks.

## Methods

### Fabrication of piezoelectric generator

PVDF-TrFE (70–30 mol%) copolymer powder and dimethylformamide (DMF) were purchased from Piezotech and Sigma Aldrich, respectively. The PVDF-TrFE copolymer powder was dissolved in DMF with 20% of volume content. The solution was prepared by stirring for 12 h at 35 °C. Then, PVDF-TrFE solution was spin-coated on a Au electrode deposited on a 20 mm × 20 mm PET substrate (thickness of 220 μm) at 3000 rpm for 30 s. The PVDF-TrFE films were annealed at 50 °C for 30 min on a hot plate. For the bottom and top electrodes, an Au electrode (thickness of 160 nm) was deposited by thermal evaporation. Cu tape, which was purchased from 3M Conductive Tape, was employed as separator between multilayers. A chip-type supercapacitor for energy harvesting was prepared by using CPH3225A (Seiko Instruments Inc., Japan) (Table S1). The charging-discharging voltage of the supercapacitor connected to different PVDF-TrFE films was compared under bending conditions with a full bridge. The discharge testing at a constant current of 100 nA was conducted with a Bio-Logics VSP potentiostat using a two-electrode system.

### Characterization

X-ray diffraction measurement (XRD, D/max-2500pc, Rigaku, Japan) was used to investigate the structures of PVDF-TrFE films coated on silicon substrate. The electrical output performance of the piezoelectric films was measured with an electrometer (Keithley 6514, Keithley Instruments Inc., USA). The capacitance was measured with a potentiostat (Model 263A, EG&G, USA). A bending machine (JIBT-210, Junil Tech, Republic of Korea) was used for bending tests under controlled conditions.

## Supplementary information

Supplementary Information_Enhanced output performance on LbL multilayer PVDF-TrFE piezoelectric films for charging supercapacitor
