# Supplementary material for: Enhanced output performance on LbL multilayer PVDF-TrFE piezoelectric films for charging supercapacitor
Source: Sci Rep. 2019 Apr 29;9:6581. doi: 10.1038/s41598-019-43098-6 (PMC6488593; doi:10.1038/s41598-019-43098-6)
Supplement: Supplementary file 1 — Supplementary Information_Enhanced output performance on LbL multilayer PVDF-TrFE piezoelectric films for charging supercapacitor [file 41598_2019_43098_MOESM1_ESM.docx]

**Supplementary Information**

**Enhanced output performance on LbL multilayer PVDF-TrFE piezoelectric films for charging supercapacitor**

Moon Hyun Chung^1,2^, Seunghwan Yoo^1,2^, Hyun-Jun Kim^1^, Jungjoon Yoo^3^, Seol-Yee Han^1^, Kyung-Hwa Yoo^2^, and Hakgeun Jeong^1*^

^1^ Energy ICT·ESS Laboratory, Energy Efficiency Technologies and Materials Science Division, Korea Institute of Energy Research, 152 Gajeong-ro, Yuseong-gu, Daejeon 34129, Republic of Korea.

^2^ Department of Physics, Yonsei University, 50 Yonsei-ro, Seodaemun-gu, Seoul 03722, Republic of Korea.

^3^ Separation and Conversion Materials Laboratory, Energy Efficiency Technologies and Materials Science Division, Korea Institute of Energy Research, 152 Gajeong-ro, Yuseong-gu, Daejeon 34129, Republic of Korea.

___________________________________________________________________________
*Corresponding author. Fax: 82 42 879 3840. Email address: hgjeong@kier.re.kr (H.G Jeong).

**Table S1** Specifications of chip-type supercapacitor, which is commercially available and was purchased from Seiko Instruments Inc. (Japan).

| Model | CPH3225A (Seiko Instruments Inc., Japan) |
| --- | --- |
| Capacitance | 11 mF |
| Internal Impedance | 160 Ω |
| Thickness | 0.9 mm |
| Body Size | 3.2 mm × 2.5 mm |
| Max. Use Voltage | 3.3 V |

**
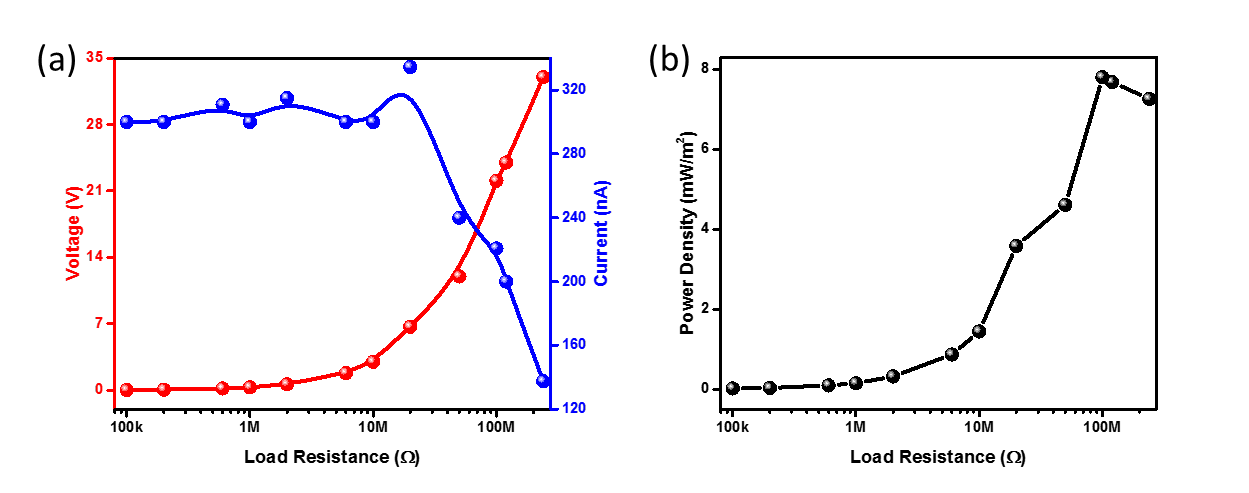
**

**Figure S1.** The output performance of LbL multilayer with different load resistance (a) the value of output voltage and current with different load resistance and (b) calculated the output power density at different load resistance.

We measured the LbL multilayer of extrinsic load resistance. Values of voltage and current can reach the 33 V and 310 nA, respectively. The power density is also conducted the variety of external load resistance. The peak power density shows 7.8 mW/m^2^ at 100MΩ.
